# Study protocol: HipSTHeR - a register-based randomised controlled trial – hip screws or (total) hip replacement for undisplaced femoral neck fractures in older patients

**DOI:** 10.1186/s12877-020-1418-2

**Published:** 2020-01-21

**Authors:** Olof Wolf, Pontus Sjöholm, Nils P. Hailer, Michael Möller, Sebastian Mukka

**Affiliations:** 1 Department of Surgical Sciences, Orthopaedics, Uppsala University, Uppsala, Sweden; 2 Department of Surgical and Perioperative Sciences (Orthopaedics), Umeå University, Umeå, Sweden; 3 Institute of Clinical Sciences, Sahlgrenska Academy, University of Gothenburg, Gothenburg, Sweden

**Keywords:** Hip fracture, Femoral neck fracture, Internal fixation, Hip arthroplasty, Register, Register-based randomised controlled trial

## Abstract

**Background:**

Femoral neck fractures (FNFs), which are common in the older population, are associated with high mortality and morbidity. Some 20% of FNFs are undisplaced (uFNFs). The routine surgical procedure for uFNFs is internal fixation (IF) with 2–3 screws/pins with a reported reoperation rate in older patients (age ≥ 75 years) of up to 21%. The reoperation rate for hemiarthroplasties for displaced fractures is lower than for undisplaced fractures operated with IF. This study will aim to determine whether the outcome for older patients with an uFNF can be improved by replacing the hip instead of preserving it.

**Methods:**

A national multicentre, register-based, randomised controlled trial (rRCT) will be conducted. For this trial, 1440 patients, ≥75 years with an acute uFNF, will be allocated. Eligible patients will be identified by the Swedish Fracture Register (SFR) platform, which will notify the admitting orthopaedic surgeon of eligibility. After informed consent has been given and documented, patients will be randomised to either IF (control group) or arthroplasty (intervention group) within the SFR platform. Injury mechanism, fracture classification, date of injury, and type of treatment are registered in the SFR. Type and brand of arthroplasty, surgical approach, and fixation are obtained from the Swedish Hip Arthroplasty Register (SHAR). The study cohort from the SFR will be cross-checked with the National Patient Register and the SHAR for outcome variables at 2, 5, and 10 years.

The primary outcome will be a composite variable comprising reoperation rate and mortality at 2 years postoperatively. Secondary endpoints will include reoperation rate and mortality as stand-alone variables. In addition, secondary endpoints will be patient-reported outcomes as measured by the Short Musculoskeletal Functional Assessment questionnaire at 1 year postoperatively as routinely collected within the SFR. Further secondary endpoints will include the occurrence of adverse events such as pneumonia, stroke or myocardial infarction and evaluation of the external validity of the study.

**Discussion:**

This large, multicentre, register-based randomised controlled trial could potentially shift the treatment of uFNFs in older patients towards primary hip arthroplasty in order to improve the outcome.

**Trial registration:**

The trial is registered at www.clinicaltrials.gov (NCT03966716); May 29, 2019.

## Background

Hip fractures are a major cause of injury, morbidity and death in older patients. Undisplaced FNF (uFNF) are classified according to the Garden classification system on the anteroposterior X-ray [1, 2]. The routine surgical procedure for uFNFs, regardless of the patient’s age, is internal fixation (IF) with 2–3 screws or pins. Advanced age has been described as a risk factor for healing complications in uFNFs [3, 4]. In older patients reoperation rates ranging between 8 and 21% have been reported in the literature [2, 5–7]. In older patients displaced FNFs are treated with hip arthroplasty, which produces better and more predictable results compared with IF [5]. The reoperation rate for hemiarthroplasties for displaced FNFs is lower than for undisplaced fractures that are operated with IF [5, 8, 9]. A randomised controlled trial (RCT) comparing modern hemiarthroplasty with screw fixation for uFNFs found no significant difference in hip function but hemiarthroplasty led to improved mobility and fewer major reoperations [8].

In the Swedish Fracture Register (SFR) all fracture types in adults and all long-bone fractures in children are registered [10]. The SFR is a unique national quality register as it contains information on fractures, regardless of treatment (surgical or non-surgical). Seventy-five per cent of the hospitals in Sweden that manage fractures on a regular basis participate in the SFR.

A question often arises as to whether the results from an RCT can be extrapolated from the study environment to a general health care setting [11, 12]. Active participation of patients and recruitment of large sample sizes in an RCT are not easily achieved and refusal to participate and loss to follow-up (a form of selection bias) are prevalent problems. Conducting register-based RCTs (rRCTs), which include a randomisation module in a large, all-inclusive clinical register with unselected consecutive enrolment, can combine some of the most important features of a prospective randomised trial with the inclusiveness and efficiency of a large-scale clinical register. The consecutive enrolment in combination with patient identification and automated linked register-based follow-up allows for a cost-effective model with analysis of those who are lost to follow-up [13].

There are limited data comparing IF and hip arthroplasty for uFNF fractures and trials have been called for to optimise the surgical treatment [2, 14].

### Aim

This study will aim to compare IF and hip arthroplasty for uFNF in the older patient using a composite variable consisting of reoperation rate and mortality. Patient-reported outcome measures (PROMs) and the frequency of adverse events will also be assessed.

## Methods/design

### Trial design, settings, and location

Hipsther is a multicentre, register-based, RCT of 1440 older patients who have sustained an uFNF. The study will be carried out from 2019 to 2029 (inclusion period 2019–2022). Patients ≥75 years with an uFNF will be identified by the registration platform. The admitting orthopaedic surgeon at a participating hospital will receive an alert about the eligibility of the patient. After screening has been performed and patient consent obtained, randomisation to either IF or arthroplasty will be carried out within the platform of the SFR before surgery. The guidelines of Good Clinical Practice (GCP-ICH) will be followed. The trial is initiated, designed, and performed as an academic investigation and registered at ClinicalTrials.gov (NCT03966716). The trial will follow the guidelines of the CONSORT (Consolidated Standards of Reporting Trials) Statement.

### Study subjects and eligibility criteria

All patients with a nondisplaced (Garden 1–2) FNF who are admitted to the participating hospitals will be screened for participation in the study. Screening will be performed during the registration in the SFR before surgery after admission to the hospital. The inclusion criteria are an acute (within 72 h) undisplaced or minimally displaced FNF (Garden 1–2) [1], age ≥ 75 years, eligible for IF or hip arthroplasty, and obtained written informed consent. Patients with dementia can be included after consent from a next of kin or power of attorney. Patients with stress or with pathological and peri-implant FNFs are excluded. In the event of bilateral uFNF during the study period only the first hip will be included and randomised.

### Randomisation and blinding

Randomisation will be computer generated and part of the research platform in the SFR. Patients are identified as eligible in conjunction with registration of demographic data and fracture pattern. In participating hospitals, the platform will alert the admitting orthopaedic surgeon about the possibility to include the patient in the study. The inclusion criteria will be stated (uFNF, ≥75 years, and fracture < 3 days old) and the treating surgeon will be asked whether the patient is eligible for inclusion and whether consent has been obtained. After screening and inclusion, the patient will be randomised within the SFR platform to receive either IF (with 2–3 screws or pins) or arthroplasty. The randomisation procedure has to be done before the patient is taken to the surgical theatre to prepare for the elected procedure. There will be no blinding given to treatment assignment. The steering committee will have no access to treatment comparisons in accumulated data until the database is locked.

### Surgical intervention

#### Hip Arthroplasty

Hip arthroplasty (hemi- or total) is performed according to the present routine at the participating hospitals. Patients > 80 years of age, cognitive dysfunction, low demand or short remaining life expectancy are normally managed with hemiarthroplasty. Patients between 60 and 79 years of age, high level of activity, and osteoarthritis or rheumatoid arthritis of the fractured hip are routinely managed with total hip arthroplasty (THA). The final decision of whether to perform a hemiarthroplasty or THA is ultimately determined by the treating surgeon. Type and brand of arthroplasty, surgical approach and method of fixation are based on the preferences of the treating surgeon and department.

#### Internal fixation

IF will be carried out with the patient on a fracture table and with the aid of an image intensifier using two cannulated screws/pins or a conventional sliding hip device. Thromboprophylaxis and antibiotic prophylaxis will be administered to patients as per hospital routine.

### When and how to withdraw patients from the trials

Should a patient request or decide to withdraw from the study, all efforts will be made to complete and report the observations as thoroughly as possible up to the point of withdrawal. For those patients who withdrew, the last post-baseline observation will be carried forward. In a case of withdrawal of full consent, the patient will be followed according to the routine standard follow-up of patients at the responsible institution but excluded from further analysis.

### Outcome measurements

#### Primary endpoint

The primary outcome of this rRCT will be a composite variable (reoperation rate and mortality). Arthroplasty is a more elaborate surgical procedure that might affect peri- and postoperative short-term mortality, as related to longer operating times, higher blood loss, and the occurrence of bone cement implantation syndrome. A higher mortality in one study group would affect the reoperation rate in that group. Therefore, we decided to construct a composite variable as the primary outcome. There will be no formal follow-ups in addition to the local clinical routines. Data on mortality and reoperations are registered in the National Patient Register (NPR) (all codes for procedures and diagnoses from hospitals), the Swedish Hip Arthroplasty Register (SHAR) (change of implant, reoperation due to dislocation, fracture or infection) and in the SFR (fracture close to implant, removal of pins/screws, conversion to arthroplasty).

#### Secondary endpoints

Secondary endpoints will include reoperation rate and mortality as stand-alone variables.

PROM, as measured by the Short Musculoskeletal Functional Assessment (SMFA) questionnaire [15], will be routinely collected within the SFR 1 year after the injury and compared with the results obtained by recall technique at the time of the fracture. No additional PROMs will be collected.

Other secondary endpoints will include the occurrence of adverse events defined as suffering, physical harm or disease, as well as death related to the index admission and as a condition that was not an inevitable consequence of the patient’s disease or treatment. The external validity of the trial will be evaluated by comparing the outcome between those who were included in the rRCT with those who fulfilled the inclusion criteria but declined to participate.

### Data collection and follow-up

Data on baseline characteristics and outcome variables are registered in the NPR, SHAR, and SFR. Follow-up will be performed postoperatively after 2, 5, and 10 years by linking between the registers (Table 1).

**Table 1:** Content for the schedule of enrolment, interventions, and assessments

TIMEPOINT	STUDY PERIOD				
	Enrolment	Allocation	Post-allocation		
	*0*	0	*2y*	*5y*	*10y*
ENROLMENT:					
Eligibility screen	X				
Informed consent	X				
Randomization		X			
INTERVENTIONS:					
*Internal Fixation (control)*		X			
*Hip Arthroplasty (intervention)*		X			
ASSESSMENTS:					
*Baseline*	X	X			
*Primary outcome*			X	X	X
*Secondary outcome*			X	X	X

Primary outcome is a composite variable of reoperation rate and mortality

Secondary outcomes are: Reoperation rate, Mortality, PROM, Adverse Events, External validity

All outcomes will come from Registers

### Data quality assurance

The study progress and conduct will be monitored by a study coordinator before, during, and after the study concludes to ensure that all aspects of the protocol are adhered to in accordance with ICH-GCP guidelines and regulatory requirements. The register data will be automatically transferred to a study data base. Because the patient has a general right to protection against invasion of privacy, each patient will receive a unique identification number. The data will then be blinded correspondingly in all data analyses. However, the study monitor, auditor, representatives from any regulatory authority, as well as the appropriate ethical committee are permitted to review the patients’ data.

### Estimated sample size and power

The reoperation rate for hemiarthroplasty due to FNF is estimated at 5% according to reports from the SHAR [16, 17]. In recent registry based studies, patients with an FNF treated with THA had a significantly reduced risk of reoperation when compared with patients treated with hemiarthroplasty [18, 19]. Because we know that the variable “reoperations” does not have 100% completeness in the SHAR given that some procedures (e.g., debridement and irrigation or closed reduction) are not reliably registered, we assume that the actual reoperation rate is closer to 7.5%. The reported reoperation rates for uFNFs vary [6, 7, 20, 21], but we estimate that the rate is about 12.5% at 1 year after IF with screws/pins in older patients with uFNF. Mortality rates are reported from 11% at 1 year in a study with both displaced and undisplaced FNFs [22] to just over 20% in other studies [6, 8, 21].

In our primary outcome composite variable (reoperation rate and mortality), we expect a 15% 1-year mortality within the study cohort. We assume that death or reoperation would occur within 1 year in 27.5% (15% + 12.5%) of the patients in the control group (IF) and aim to detect a decrease to 22.5% (15% + 7.5%) in the intervention group (arthroplasty). We plan to include patients for 3 years. Patients will be censored at the time of reoperation or at time of death within 2 years, at time of withdrawal of informed consent or at the end of the study. The end of study will be when the last patient has a 1-year follow-up, cross-checking with the NPR has a 1 year delay for data completeness; hence, about two thirds of the patients will have a 2-year follow-up and the remaining patients between 1 and 2 years. Simulations under an assumption of constant hazard indicate that 1440 patients enrolled for 3 years, yielding about 586 events, would produce a power of 80% to detect such a difference. The analyses were performed using R v. 3.3.1 and the survival package v. 2.39.4.

### Statistics

Time to first event will be presented as Kaplan-Meier plots. The primary composite and the individual components will be analysed for time to first event using Cox regression adjusted for age as a linear covariate and presented as hazard ratios with 95% confidence intervals and *p*-value. Because of the register follow-up, we assume that follow-up will be complete. In the rare event that a patient is known to have incomplete follow-up, the patient will be considered censored at the last known follow-up. For the reoperation endpoint, death will be handled as censoring events.

The number of patients with a perioperative event and event within 2 years will be summarised in tables. Patients lost to follow-up will be included in the denominator, and the 2-year frequencies will only include patients randomised at least 2 years before data collection. Supplementary sensitivity analyses will include analyses censored at 1 year and analyses of the number of patients with events within 2 years. Secondary outcomes will be presented in the same way as the primary composite and its components.

Statistical expertise at the Uppsala Clinical Research Center will perform the statistical analyses. Data will be blinded for the principal investigators until the final analysis has been performed and until the writing of the manuscript.

### Ethics and dissemination

The study will be conducted in accordance with the ethical principles of the Declaration of Helsinki. The study has been approved by the Swedish Ethical Review Authority (Dnr: 2019–00140). Any protocol amendments will be approved by the Swedish Ethical Review Authority and published at www.clinicaltrials.gov (NCT03966716). The first results from the study will be circulated to the medical community through presentations and publications in a scientific, peer-reviewed, open access medical journal.

## Discussion

The present trial will provide improved evidence for the future choice of treatment for patients with Garden 1–2 FNF who are ≥75 years old.

Patients with dementia represent a large proportion of the hip fracture population but the inclusion of this group is difficult because of the need of informed consent by next of kin (or power of attorney) and thus exposes the study for inclusion bias. Informed consent has been constructed in two versions, one for lucid patients and one for patients with cognitive impairment not able to give informed consent; the latter version allows us to address next of kin. There is a national effort in Sweden to treat hip fractures within 24 h of admission and with operative intervention performed only during duty hours, the recruitment process is difficult, which could result in selection bias. The Garden classification does not take into account posterior or anterior fracture tilt. There are reports discussing the linkage of posterior and anterior tilt to failure after IF, a situation that might lead to reluctance to include these patients in this study [20, 21, 23–25].

The primary endpoint, defined as a composite variable (reoperation rate and mortality), is constructed to consider both mortality and reoperations. A longer and more strenuous initial procedure (i.e. arthroplasty) might affect peri- and postoperative mortality and thus mask potential reoperations. However, a recent RCT comparing hemiarthroplasty and IF in patients with uFNF reported a higher mortality rate after IF [8].

The external validity of the trial will be evaluated by comparing mortality, complications, and PROM between those who were included in the rRCT, those who fulfil the inclusion criteria for the rRCT trial but declined participation, and those eligible but not screened. The limitations of the present trial include the lack of blinding of surgeons and patients, the potential of randomisation bias because the two compared procedures (IF, arthroplasty) differ in complexity, required surgeon skills, and duration. The strengths of the study are the pragmatic rRCT study design that includes a national coverage of hospitals, a large sample size, and clinically relevant outcomes. The results from the study will be dispersed to the medical community by way of presentations and publications in relevant medical journals.

## Data Availability

The datasets used during the current study are not publicly available because of patient integrity but are available from the corresponding author on reasonable request.

## Abbreviations

IF: internal fixation
NPR: National Patient Register
PROMs: patient-reported outcome measures
rRCT: register-based randomised controlled trial
SFR: Swedish Fracture Register
SHAR: Swedish Hip Arthroplasty Register
SMFA: Short Musculoskeletal Functional Assessment questionnaire
uFNF: undisplaced femoral neck fracture
